# A Case of Epidural Management in a Pregnant Patient with Spinal Epidural Lipomatosis

**DOI:** 10.7759/cureus.5553

**Published:** 2019-09-02

**Authors:** Haider Ali, Jeff Huang

**Affiliations:** 1 Anesthesiology, University of Central Florida College of Medicine, Orlando, USA

**Keywords:** spinal epidural anesthesia, spinal epidural lipomatosis, neuraxial anesthesia, epidural, epidural lipomatosis, labor epidural, spinal anesthesia, epidural analgesia, epidural anesthesia

## Abstract

Spinal epidural lipomatosis (SEL) is a rare condition in which fat accumulates in the epidural space. Patients may be asymptomatic or present with neurologic symptoms if the spinal cord is compressed. Based on the pathophysiology of this condition, there are theorized effects that suggest a contraindication to epidural anesthesia. Due to the rarity of the condition, there is a lack of significant evidence in the literature regarding the efficacy of epidural analgesia or related complications in these patients. Herein, we have presented a patient with a prior diagnosis of spinal epidural lipomatosis and her course of anesthetic management during labor.

## Introduction

Spinal epidural lipomatosis (SEL) is an uncommon condition characterized by an abnormal amount of fat accumulated in the epidural space [1-3]. In this disease process, fat deposits on or near the spinal cord have the capacity to cause neurologic deficits via compression [4-6]. Although SEL may be idiopathic, the most common cause of SEL is attributed to the use of exogenous steroids in a dose-dependent manner [2,6].

Prior literature suggests that repeated failure of epidural analgesia, with large volumes of anesthetic, may be suggestive of the existing epidural lipomatosis, indicating that there may be a contraindication to anesthesia in patients with known epidural lipomatosis [7]. It has been hypothesized that epidural fat accumulation may affect the efficacy of delivered anesthetics, as segmental conduction blocks have been observed in these situations [7]. Herein, we present a case of SEL diagnosed before pregnancy and the patient’s labor analgesia management.

## Case presentation

**Figure 1:**
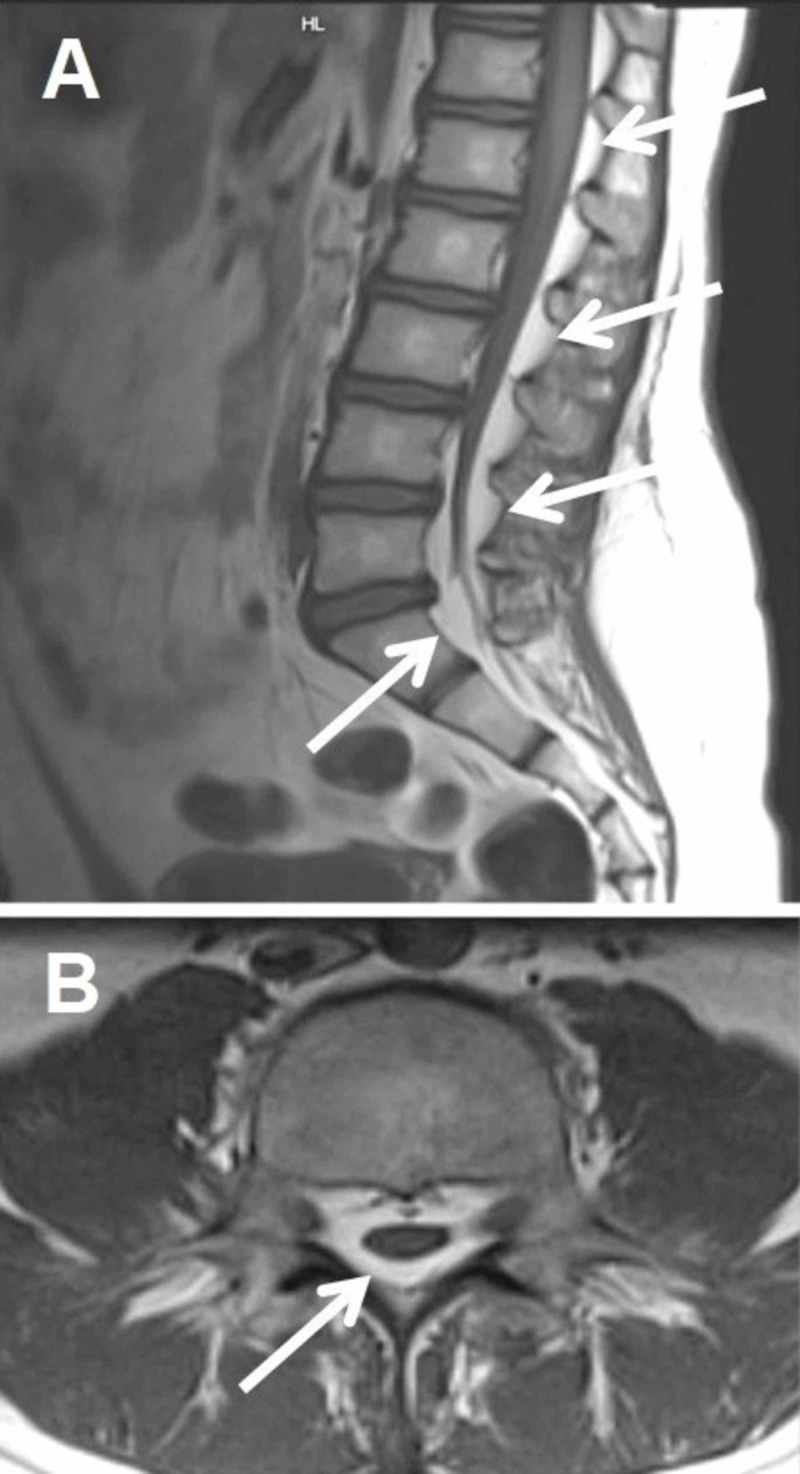
A sample MRI of the lumbar spine, demonstrating spinal epidural lipomatosis The arrows point to selected areas of the pathological accumulation of fatty tissue in the epidural space. T1 weighted images: A, sagittal plane; B, axial plane. From [8], with permission under the terms of the Creative Commons Attribution License.

A 22-year-old, gravida 1 para 0, was admitted to the Winnie Palmer Hospital for Women & Babies in Orlando, FL at 35.4 weeks for induction of labor. The patient was diagnosed with SEL in 2016. Figure 1 shows the MRI of the lumbar spine, demonstrating SEL.

The patient's past medical history includes endometriosis, SLE, hypothyroidism, tachycardia/bradycardia syndrome status post pacemaker insertion, supraventricular tachycardia, postural orthostatic tachycardia syndrome, endocarditis with sepsis, coronary aneurysms, pericarditis, tricuspid/pulmonary regurgitation, seizure disorder, and complex migraines. Her surgical history includes two pacemaker surgeries, four cardiac catheterizations, radical laparoscopy for endometriosis, two transesophageal echocardiograms (TEE), and a Hickman line placement. Her active medications include prednisone 10 mg qd, atenolol 50 mg qd, hydroxychloroquine 200 mg q12 hours, and prenatal vitamins.

Her airway, cardiovascular, and pulmonary examinations were normal. A neurologic examination of cranial nerves, sensation, motor function, cerebellar function, and reflexes was unremarkable. An echocardiogram determined a left ventricular ejection fraction of 65-69% and showed no evidence of pericardial effusion. A CT scan of her chest was unremarkable. The patient was educated on the risks and benefits of neuraxial anesthesia based on the available literature.

Upon evaluation, the risks were predicted to be only slightly elevated compared to a routine labor epidural. However, no further risk quantiﬁcation could be established due to the rarity of the patient’s condition.

The patient requested epidural analgesia and a lumbar epidural catheter was placed at the L3-L4 interspace in one attempt. The patient received adequate pain control with the standard epidural infusion of 0.1% ropivacaine with 2 mcg/ml Fentanyl at 10 ml/hour with patient-controlled boluses of 10 ml every 20 minutes and a maximum dose of 3 boluses per hour. The patient had an uncomplicated delivery and an uneventful hospital stay.

## Discussion

SEL is a relatively rare condition characterized by a variable, but an abnormal amount of fat accumulated in the epidural space. Although SEL may be idiopathic, the most common cause of SEL is attributed to the use of exogenous steroids, in a dose-dependent manner [2,6]. Other secondary causes include Cushing’s syndrome, use of protease inhibitors, and obesity [2-3]. SEL is reported to be underdiagnosed, especially with the rise of obesity, which is a significant risk factor for SEL [9].

In individuals with this condition, the deposited adipose tissue may or may not compress the spinal cord, which could produce neurological symptoms [4-6]. Regardless of the presence of symptoms, there is a reasonable concern for epidural administration due to the theorized effect of the abnormal tissue on medication delivery and distribution. This may be due to the need for multiple puncture attempts, increasing the risk of spinal cord-related complications [7].

An example of a case in prior literature describes a patient where a small dural sac was discovered due to the increased content of epidural fat [10]. The relevant aspects of providing spinal anesthesia were discussed in the paper, describing the expected anatomical complications due to the disease process. This was theorized based on the lack of spinal fluid return, leading to multiple puncture attempts and thus increasing the risk of nerve damage.

The effects and potential complications likely depend on the variation of distributed tissue; however, successful cases have rarely ever been reported to allow providers to cautiously provide pain management for patients with a need for epidural analgesia. One such prior case has been reported in which a pregnant patient with a prior diagnosis of SEL underwent an epidural anesthetic procedure before labor [11]. There were no challenges during the administration of epidural anesthesia nor subsequent relevant complications. The therapy was found to be efficacious for its intended purposes during labor and delivery, just as in the case we present.

These cases provide needed evidence for safe epidural administration for patients with diagnosed SEL. The anesthetic management for these patients, however, should still be approached cautiously, given the variability of the disease process of SEL and how it may manifest from patient to patient.

## Conclusions

Epidural anesthesia is theorized to be contradicted in patients diagnosed with SEL; however, evidence for safe epidural administration in these patients is lacking due to the rarity of SEL. In the case presented, a patient with a prior diagnosis of SEL received successful epidural analgesia without any challenges or consequent complications. Given the variability of the disease process, anesthetic management for patients with SEL should still be approached cautiously and assessed individually. 
